# The Association Between Changes in the University Educational Setting and Peer Relationships: Effects in Students' Depressive Symptoms During the COVID-19 Pandemic

**DOI:** 10.3389/fpsyt.2021.783776

**Published:** 2021-12-14

**Authors:** Virgínia Conceição, Inês Rothes, Ricardo Gusmão

**Affiliations:** ^1^The Epidemiology Research Unit–Institute of Public Health, University of Porto, Porto, Portugal; ^2^Laboratory for Integrative and Translational Research in Population Health, Porto, Portugal; ^3^Faculty of Psychology and Education Science, University of Porto, Porto, Portugal; ^4^Center for Psychology at University of Porto, Porto, Portugal

**Keywords:** COVID-19, depression, anxiety, online teaching, help-seeking behaviors

## Abstract

**Objective:** Abrupt life changes imposed by the lockdown measures, with a direct impact on teaching methodology and social interactions, as well as sleeping patterns, harmed university students' mental health. This study aimed to analyze the relationship between satisfaction with online teaching, social interaction with depression, anxiety symptomatology, and to analyze the effects of the pandemic and the lockdown in mental care access.

**Methods:** The online survey collected demographic data, satisfaction with online teaching, and social interaction. We evaluated the depression and anxiety symptomatology using the 9-item Patient Health Questionnaire and the 7-item Generalized Anxiety Disorder, respectively. For the PHQ-9, we used the cut-off 15 for moderately severe depressive symptoms, whereas for GAD-7, we recurred to the cut-off 10 for moderately severe anxiety symptoms. This study used three data points: October 2019, June 2020, and March 2021.

**Findings:** The study included *n* = 366 participants from all university study fields, with a mean age of 21.71 (SD = 1.42) in the last survey, and 71.3% were women. Depressive symptoms increased significantly from October 2019 to June 2020, and the mean scores grew until March 2021. Anxiety symptoms also significantly increased from October 2019 to June 2020; however, from June 2020 to March 2021, there was a non-significant decrease in the proportion. Mean scores for satisfaction with online teaching were 38.23% in June 2020 and 34.25% in March 2021, a non-significant difference. Satisfaction with social interaction significantly decreased from 37.35% in 2020 to 24.41% in 2021. Participants with scores above the cut-off of moderately severe and severe depressive and anxiety symptoms showed significantly lower satisfaction with online teaching than students with lower depression and anxiety scores. Despite the significant increase in clinical symptomatology, help-seeking behaviors did not change accordingly, and more than 50% of the students with mild or severe depressive and anxiety symptomatology did not get treatment during the pandemic.

**Conclusion:** The findings of this study suggest that most students are dissatisfied with online teaching and the type of social interaction they were forced to adopt because of the pandemic. The severity of depressive and anxiety symptomatology significantly increased between October 2019 and March 2021, but help-seeking behaviors did not increase accordingly.

## Introduction

The COVID-19 pandemic had a massive impact on public life, and for the first time in recent history, a large set of restrictions changed life as we used to know. In Portugal, as in most countries, we experienced the interdiction of face-to-face interactions, the closing of all schools and universities, and a vast set of adjustments to our lifestyle. These changes affected everyone with no exception; however, university students became a critical risk group because of the already high reported rates of mental illness among university students (1).

It soon became clear that the quarantine strategies harmed students' mental health alongside a consensus on the literature about increased anxiety and depression symptomatology (2–4). Previous studies have described increments in fear (2, 3), worry (4), and stress (5). However, most of the research is still cross-sectional or without pre-pandemic information in the same sample (5–8), and the maintenance of the symptoms after the quarantine is also not straightforward in the literature (9).

University students have also reported sleep alterations during the pandemic, with an evident decrease in sleep quality (10), insomnia and other sleep alterations (11).

Research on the effects of the lockdown in Europe in university students is concordant regarding the harm on mental health, especially concerning depressive and anxiety symptomatology (9, 12–14).

The most recent study, published by Kohls et al. (14) concluded that university students are a vulnerable risk for the development of mental illness as a result of the lockdown, namely depression. Another important conclusion of this study is the evidence that only half of the students diagnosed with any mental disorder received treatment.

Before the pandemic, Paul et al. (15) were interested in comparing traditional and online teaching methods: focusing on student performance, they found no differences between online and face-to-face students' performance overall between gender or class rank.

Students' learning experiences and the effectiveness of online programs are some of the challenges of online teaching (16), and student satisfaction, along with outcomes, can be a good indicator of the quality of the programs (17). To the best of our knowledge, not many studies investigated student satisfaction with online teaching. Most of them included students from the health care areas (16, 18, 19), and results are far from consensual. The Rajabalee and Santally study (16) showed that students were generally satisfied with the online learning experience and performance levels. On the other hand, they reported low levels of satisfaction with tutor support and technical difficulties. Dutta et al. (18) concluded, in their study with medical and nursing students, that online teaching is not an effective alternative. Rota et al. (19), evaluating professors' perceptions about online teaching, depicted that most academics favored providing online teaching, but opinions were almost unanimous that distant education could not substitute face-to-face teaching.

Due to the expected consequences of the pandemic and its life-change implications, many countries started to promote the importance of mental health care, and virtual care has become a growing area of investment (20, 21). Previous studies on mental health care help-seeking behaviors have established that many university students with mental health problems do not seek help (22). The most frequently identified help-seeking barriers are stigma and embarrassment about help-seeking and poor mental health literacy (23–25).

Even though many institutions invested many resources in developing mental health care programs remotely accessible, there is a lack of information on how people adjusted in terms of help-seeking behaviors.

With our study, we aimed (a) to evaluate the impact of COVID-19 lockdown in university students' anxiety and depression symptoms and how it progressed during the pandemic; (b) to analyze self-reported changes in satisfaction with online teaching, social interaction, and sleeping changes; and (c) to evaluate the effects of the pandemic in mental care access.

## Materials and Methods

### Participants

The current study used three surveys: October 2019 (before the pandemic), June 2020 and March 2021 (during the pandemic). We extracted participants' data from a cohort of students from the University of Porto. The data was collected throughout an online survey and included participants registered in the first year from all University courses in 2019. More detailed information about this cohort participants is available elsewhere (12, 26).

The context of the surveys in 2020 and 2021 was considerably different in terms of lockdown measures. In 2020, lockdown had started on the 18th of March, and in June, lockdown measures were softer: shopping centers, cinemas, theaters, and gyms were open, however with restrictions in their maximum capacity of people and closing time; outside gatherings were allowed for a maximum of 20 people. In 2021, a new lockdown started in January, and lockdown measures were still stringent in March, with minimal face-to-face interactions allowed: civic duty of home collection, prohibition of events or gatherings with more than 10 people, most of the shops were closed, cinemas, theaters and gyms were also closed. Both in 2020 and 2021 surveys, universities were still closed, and teaching was exclusively online.

### Procedures and Outcomes

We asked participants to answer a short socio-demographic questionnaire about sex, age, and previous mental health care. In the 2020 and 2021 surveys, we also asked if students knew anyone infected by COVID-19 and if they were infected.

Participants also answered the Portuguese versions of The Patient Health Questionnaire (PHQ-9) (27, 28) and the Portuguese version of the Generalized Anxiety Disorder (GAD-7) (28) in all three surveys. Cronbach's alpha of the PHQ-9 was 0.86 at baseline, and GAD-7's Cronbach alpha was 0.91, indicating good internal reliabilities.

We analyzed PHQ-9 and GAD-7 scores as continuous variables, indicating a central average score for the sample and a binary threshold score, indicating the proportion of participants with a clinically significant level of symptoms, at least moderate, in need of assessment and possibly, intervention. The cut-off point for moderate symptomatology on the PHQ-9 scale is 15 (29) and ten on the GAD-7 scale (30).

### Data Analysis

Data were analyzed with a 95% confidence interval, using SPSS 24.

We used Student's *t*-test to compare groups in continuous variables and the Mann-Whitney test to compare proportions between two groups. We explored the differences in PHQ-9 and GAD-7 scores across time, using One-Way ANOVA repeated measures to assess changes in means with *post-hoc* Bonferroni comparisons and Cochran's Q test with McNemar's *post-hoc* Bonferroni-adjusted alpha to evaluate the changes in cut-off proportions between the different surveys. We also calculated the partial eta square to understand the effect of time in the depressive and anxiety symptomatology scores across time.

### Ethical, Registration, and Guidelines Considerations

This study comprises comprehensive longitudinal research on first-year university students' mental health, including an experimental single-blind randomized control trial.

(ISRCTN970936), moreover registered as an observational study to analyze the effects of COVID-19 in this cohort (ISRCTN63459073).

It complies with the relevant national and institutional committees' ethical standards on human experimentation and the Helsinki Declaration of 1975, as revised in 2008. The Institute of Public Health of the University of Porto ethics committee approved the research with the ID reference CE18096. All participants signed an informed consent digital form according to the Helsinki and Oviedo Conventions.

To avoid possible inadequacies in the study reporting, we followed the Strengthening the Reporting of Observational Studies in Epidemiology (STROBE) (31) guidelines in the construction and preparation of the study.

## Results

### Participants

In October 2019, our cohort included 623 participants. In June 2020, the number of participants decreased to 401, and in March 2021, 366 participants answered our questionnaire. Our sample included the 366 participants with answers in all of these three surveys.

In the last survey, participants' mean age was 20.71 (SD = 1.42), and 71.3% (*n* = 261) were women and came from all 14 schools of the University of Porto.

Compared with the sample in October 2019, the participation rate was 58.7%, but we did not observe significant differences between participants and dropouts on depressive and anxiety symptomatology. In October 2019, the 366 participants included in the final sample presented a total score of 9.53 (SD = 3.27) on the depressive symptomatology scale, while the dropouts (*n* = 257) showed a mean score of 9.57 (SD = 3.56) [*t*_(2, 621)_ = −0.07, *p* = 0.95]. On the anxiety symptomatology scale, at the moment of the first survey, included participants obtained a mean of 9.74 (SD = 4.25), and dropouts showed a mean of 9.86 (SD = 4.05) [*t*_(2, 621)_ = −0.23, *p* = 0.82].

In March 2021, 65% (*n* = 238) of the participants knew someone infected with COVID-19, and 20.2% (*n* = 74) were infected themselves. Most of the participants (68.6%, *n* = 251) reported going to sleep later after the beginning of the pandemic, resulting in a mean of 6.77 (SD = 1.13) hours asleep per night.

### Self-Reported Satisfaction With Online Teaching and Social Interaction

**Figure 1:**
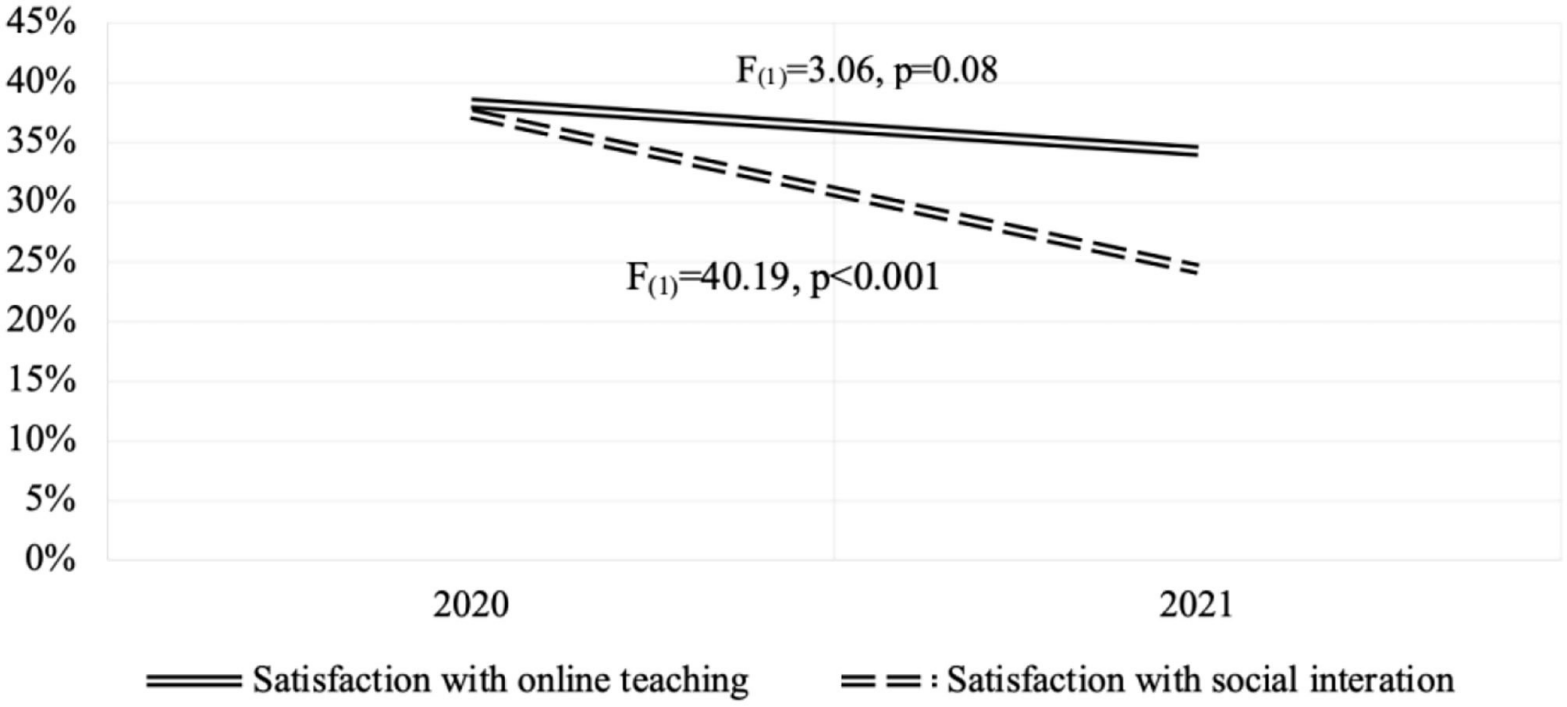
Evolution of satisfaction with online teaching and social interaction between 2020 and 2021.

Ensuing the pandemic, participants showed low satisfaction levels with online teaching, with a mean of 38.32% (SD = 25.57) in 2020 and 34.25% (SD = 29.42) in 2021. Even though we observed a decrease, the difference was not significant, as shown in Figure 1. On the other hand, satisfaction with social interaction also experienced a significant decline: a mean of 37.35% (SD = 23.83) in 2020 to 24.41% (SD = 21.08) in 2021 (Figure 1).

We did not identify a significant difference between gender and satisfaction scores over time in any measures. In satisfaction with online teaching, the effect was [*F*_(1)_ = 0.18, *p* = 0.67], and in satisfaction with social interaction was [*F*_(1)_ = 0.93, *p* = 0.34].

### Clinical Symptomatology

**Figure 2:**
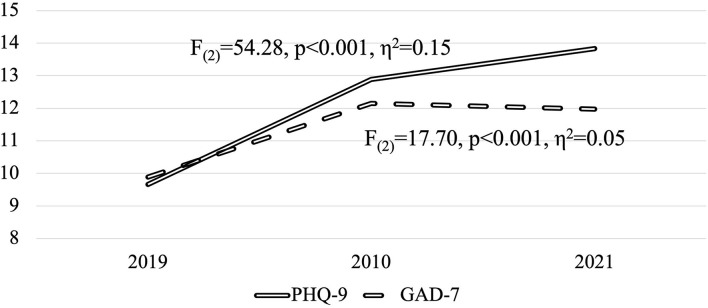
Evolution of PHQ-9 and GAD-7 mean scores between 2019, 2020, and 2021.

As we can observe in Table 1, Figure 2, the mean score of depressive symptomatology significantly increased after the beginning of the pandemic. The observed changes are significant from each time point with the other, as *post-hoc* tests showed a *p* < 0.001 in each comparison. We also observe a significant time effect, as the eta squared is higher than 0.14.

**Table 1:** Depressive and anxiety symptomatology changes across time.

		**Oct 2019**	**June 2020**	**March 2021**	**Test**	**η^2^**
PHQ-9	<15 (n)	78.4% (*n* = 287)	62.5% (*n* = 229)	51.2% (*n* = 187)	$\chi_{\left(2\right)}^{2}$ = 54.11, *p* < 0.001	0.17
	≥15 (n)	21.6% (*n* = 79)	37.5% (*n* = 137)	48.6% (*n* = 179)		
GAD-7	<10 (n)	53.9% (*n* = 287)	34.6% (*n* = 127)	40.3% (*n* = 147)	$\chi_{\left(2\right)}^{2}$ = 22.90, *p* < 0.001	
	≥10 (n)	46.1% (*n* = 79)	65.4% (*n* = 239)	59.7% (*n* = 219)		0.06

The proportion of students with moderate-severe and severe depressive symptomatology increased significantly after the pandemic and kept growing. *Post-hoc* tests using McNemar's with Bonferroni-adjusted alpha level showed significant differences in the proportion distributions between all surveys.

No significant interaction was detected between the mean scores throughout time, knowing someone infected or being infected. Nonetheless, we observed a significant effect of being infected with COVID-19 and the moderate-severe and severe depressive symptomatology in 2021, where 60.5% of those who scored 15 points or above got COVID-19 (*f* = 0.15, *p* < 0.05). This effect corresponds to an odds-ratio of developing depressive clinically relevant symptomatology of 2.18 (CI = 1.06–4.45).

Time also revealed a significant effect on anxiety symptomatology, yet there was a slight decrease in the symptomatology mean from 2020 to 2021 (Table 1). The *post-hoc* test revealed that the differences are only significant between the 2019 survey and 2020 and 2021 individually (*p* < 0.001). Between 2020 and 2021, the difference was no longer significant (*p* = 0.89). The observed eta squared indicates a medium effect of time.

We also observed a significant increase in the proportion of moderate-severe and severe anxiety symptomatology from 2010 to 2020. McNemar's with Bonferroni-adjusted alpha level *post-hoc* test did not show a significant difference between 2020 and 2021 (*p* = 0.11).

In the anxiety symptomatology, no significant interaction was detected between the mean scores nor proportions throughout time, knowing someone infected or being infected.

Participants with more severe depressive and anxiety symptoms reported less satisfaction with online teaching and social interaction (Table 2), with a significant Pearson correlation between depressive and anxiety symptomatology total score and satisfaction with online teaching and social interaction.

**Table 2:** Satisfaction with online teaching and social interaction according to depressive and anxiety symptomatology.

			**June 2020** **M (SD)**	**March 2021** **M (SD)**
PHQ-9	Satisfaction with online teaching	<15	44.0 (25.10)	37.74 (29.74)
		≥15	33.7 (27.22)	30.52 (26.56)
		Test	*t*_(364)_ = 4.88, *p* < 0.001	*t*_(364)_ = 2.68, *p* < 0.01
	Satisfaction with social interaction	<15	43.41 (23.32)	27.20 (23.86)
		≥15	30.54 (21.34)	20.26 (23.11)
		Test	*t*_(364)_ = 7.09, *p* < 0.001	*t*_(364)_ = 3.34, *p* < 0.01
GAD-7	Satisfaction with online teaching	<15	49.15 (24.80)	39.93 (29.87)
		≥15	35.41 (25.91)	29.64 (19.69)
		Test	*t*_(364)_ = 4.88, *p* < 0.001	*t*_(364)_ = 3.86, *p* < 0.001
	Satisfaction with social interaction	<15	47.28 (23.09)	28.95 (24.01)
		≥15	33.11 (22.12)	20.44 (22.06)
		Test	*t*_(364)_ = 7.99, *p* < 0.001	*t*_(364)_ = 4.03, *p* < 0.001

Along 2020 and 2021, depressive symptomatology correlated negatively both with satisfaction with online teaching (respectively *r* = −0.59, *p* < 0.001; *r* = −0.41, *p* < 0.001) and with social interaction (respectively *r* = −0.66, *p* < 0.001; *r* = −0.47, *p* < 0.001). Similarly, in 2020 and 2021, negative correlations occurred between anxiety symptomatology and online teaching (*r* = −0.47, *p* < 0.001; *r* = −0.36, *p* < 0.001) and satisfaction with social interaction (*r* = −0.49, *p* < 0.001; *r* = −0.48, *p* < 0.001).

### Help-Seeking Behaviors

As we can observe, in Table 3, there was an increase in treatment access after the pandemic, but these changes were not significant: χ2_(2)_ = 1.78 *p* = 0.4. The most significant change observed has to do with the symptomatology measured in the different periods of mental health care treatment: before the pandemic, the number of students who received treatment and did not score above the cut-off in any of the scales is considerably higher than after the pandemic. We also observe that more than half of the students with mild or severe depressive and anxiety symptomatology did not get treatment during the pandemic (62.1% in June 2020, *n* = 227, and 56.1% in March 2021, *n* = 205), a number significantly higher than pre-pandemic (48.5%, *n* = 177).

**Table 3:** Global treatment proportions throughout time and according to clinical symptomatology.

		**October 2019** **(n)**	**June 2020** **(n)**	**March 2021** **(n)**
Students who received mental health-related treatment		19.9% (*n* = 73)	22.5% (*n* = 82)	21% (*n* = 77)
Symptomatology of those who received treatment	Under cut-offs	36.5% (*n* = 134)	24.7% (*n* = 90)	23.9% (*n* = 87)
	Above cut-off in one scale	28.5% (*n* = 104)	22.7% (*n* = 83)	24.2% (*n* = 89)
	Above cut-off in both scales	35% (*n* = 128)	52.6% (*n* = 193)	52% (*n* = 190)
Symptomatology of those who did not receive treatment	Under cut-offs	51.5% (*n* = 188)	37.9% (*n* = 139)	43.9% (*n* = 161)
	Above cut-off in one scale	33.4% (*n* = 122)	30.2% (*n* = 110)	25% (*n* = 90)
	Above cut-off in both scales	15.1% (*n* = 56)	31.9% (*n* = 117)	31.1% (*n* = 115)

## Discussion

One of the leading life alterations in university students' lives was the closing of universities, which meant the learning setting and the impossibility of face-to-face interactions with peers and professors. From a research perspective and for professors and policy-makers, student satisfaction and engagement are essential in higher education (16).

In our sample, satisfaction with online teaching was low both in the 2020 and 2021 surveys. The international research on students' satisfaction with online teaching is scarce with mixed results. For instance, only 25.6% of students manifested low satisfaction with one specific e-learning module (16). On the other hand, most Indian medical and nursing students reported dissatisfaction with online teaching, with 42% of the sample reporting very dissatisfied or dissatisfied (18). Since this is an essential issue for student engagement and academic success, more research is needed to understand the factors underlying students' perspectives on the educational settings. In a study with academics from Italian universities, participants agreed that distant education could not substitute the value of learning with personal interactions (19).

Regarding satisfaction with social interaction, we observed a significant decrease from 2020 to 2021; this decline may be due to the tiredness of the students after more than 1 year of restrictions and the more restricted lockdown measures at the moment of the 2021 survey.

Regarding sleeping patterns, our sample revealed a similar result to the existing literature (10, 11), with most students reporting fewer hours asleep and going to sleep later than before the pandemic.

Depressive symptomatology significantly increased over 18 months from the pre-pandemic period throughout the second pandemic lockdown. In 2021, almost half of the sample presented moderate-severe to severe depressive symptomatology. Even though sex or knowing someone infected with COVID-19 did not significantly affect depressive symptomatology, getting the illness significantly increased the risk of depression.

We also observed a significant correlation between satisfaction with online teaching and satisfaction with social interaction and depressive symptomatology. Students who scored above the cut-off for moderately severe and severe depressive symptoms presented significantly lower levels of satisfaction with online teaching and social interaction, an expectable consequence of depression.

Anxiety symptoms also significantly increased from 2019 to 2020. Even though we see a slight decrease from 2020 to 2021, the difference was not significant and may indicate adaptation and habituation mechanisms acting as protectors and promoting student resilience. We also found no significant interaction with sex, knowing someone infected, or getting the illness.

However, correlations between satisfaction with social interaction, online teaching, and anxiety symptoms were as significant as those found for depressive symptomology. Nonetheless, participants who scored above the cut-off in the anxiety scale presented significantly lower satisfaction levels in online teaching and social interaction.

Despite the apparent mental health detriment spanning 18 months from 2019 to 2020 and 2021, the investment in the promotion of mental health care and the development of virtual educational solutions (20, 21), the number of students receiving treatment did not significantly increase after the beginning of the pandemic. The WHO mental health survey estimated that only 23.1% of the students with mental illness received adequate treatment (22). This number was obtained pre-pandemic and is higher than the one obtained in our sample in 2019. This difference may be illusory, resulting from the inclusion of Portugal in the high-income group, which may not represent the actual rate since there is still vital work to be done in terms of mental health care accessibility and availability (32).

Although the difference in the global proportion of students who received treatment did not significantly change, we observed a change in the clinical symptomatology of those who got help: the proportion of students receiving mental health treatment in 2020 and 2021 with clinically relevant symptomatology was higher than in 2019. Still, more than half of the students with mild or severe depressive and anxiety symptomatology did not get treatment during the pandemic, a number significantly higher than pre-pandemic.

One limitation of our study is the dropout rate: we lost 41.3% of our sample, increasing the risk of selection bias due to increased online activity in general and online research accrued due to the pandemic. However, there are no differences between participants, and 366 subjects is a good participant number for a longitudinal study.

One main strength of our study is that we can compare data pre and post-pandemic in the same sample, surpassing some of the limitations identified in other research, as cross-sectional data mainly was used (5–8). Also, cohort studies may minimize sampling bias and are more robust to accurately identify the effects of the COVID-19 lockdown on mental health methodology (33). Likewise, we only included participants with answers in all the evaluation moments, reducing the risk of bias.

Students from all different schools and courses of the university were included, which is a significant strength of our research. Most studies on the subject include only health sector university students.

To our knowledge, this is the first study in Portugal to evaluate the actual mental health help-care seeking in university students presenting data on treatment care before and after the pandemic.

Future research could explore further the relationship between satisfaction with online teaching, depressive and anxiety symptomatology, and academic engagements and results. It would also be interesting to examine further the evolution of clinical symptomatology after the end of the restricted confinement measures in the same sample.

## Data Availability Statement

The raw data supporting the conclusions of this article will be made available by the authors, without undue reservation.

## Ethics Statement

The studies involving human participants were reviewed and approved by Institute of Public Health of University of Porto. The patients/participants provided their written informed consent to participate in this study.

## Author Contributions

VC: conceived the main idea of the study, to the design and implementation of the research, the analysis and interpretation of the results, and led the writing of the manuscript with input from all authors. IR: contributed to the design and implementation of the research. RG: contributed to the planning and supervision of the research, analysis and interpretation of the results, and manuscript writing.

## Funding

This work was funded by FCT – Foundation for Science and Technology, IP, national funds finance this work under project UIDB / 04750/2020, and Terapiailimitada – Intervenção, Formação, Consultadoria E Investigação Em Saúde Mental, Lda, Lisbon.

## Conflict of Interest

The authors declare that the research was conducted in the absence of any commercial or financial relationships that could be construed as a potential conflict of interest.

## Publisher's Note

All claims expressed in this article are solely those of the authors and do not necessarily represent those of their affiliated organizations, or those of the publisher, the editors and the reviewers. Any product that may be evaluated in this article, or claim that may be made by its manufacturer, is not guaranteed or endorsed by the publisher.
