# Supplementary material for: The Association Between Changes in the University Educational Setting and Peer Relationships: Effects in Students' Depressive Symptoms During the COVID-19 Pandemic
Source: Front Psychiatry. 2021 Dec 14;12:783776. doi: 10.3389/fpsyt.2021.783776 (PMC8712485; doi:10.3389/fpsyt.2021.783776)
Supplement: Supplementary file 1 [file Data_Sheet_1.PDF]

Supplementary file

Table 1: Participant distribution per University Faculty

**Faculty**	**n**	**%**
Engineering	82	23.41%
Humanities	69	18.85%
Sciences	8	2.19%
Biomedical Sciences	29	7.92%
Economics	28	7.65%
Law	35	9.56%
Psychology and Educational Sciences	24	5.56%
Arts	23	6.28%
Pharmaceutical Sciences	20	5.46%
Medicine	19	5.19%
Nutrition	14	3.83%
Architecture	4	1.09%
Sports	8	2.19%
Dental Medicine	3	0.82%

Table 2: PHQ-9 scores frequency.

Score	Oct 2019 N (%)	June 2020 N (%)	March 2021 N (%)
0	25 (6.83%)	3 (0.82%)	3 (0.82%)
1	22 (6.01%)	18 (4.92%)	4 (1.09%)
2	21 (5.74%)	6 (1.64%)	8 (2.19%)
3	21 (5.74%)	23 (6.28%)	14 (3.83%)
4	20 (5.46%)	15 (4.10%)	19 (5.19%)
5	22 (6.01%)	14 (3.83%)	10 (2.73%)
6	12 (3.28%)	20 (5.46%)	9 (2.46%)
7	13 (3.55%)	10 (2.73%)	17 (4.64%)
8	21 (5.74%)	12 (3.28%)	7 (1.91%)
9	36 (9.84%)	19 (5.19%)	24 (6.56%)
10	18 (4.92%)	13 (3.55%)	10 (2.73%)
11	15 (4.10%)	13 (3.55%)	15 (4.10%)
12	10 (2.73%)	19 (5.19%)	14 (3.83%)
13	15 (4.10%)	28 (7.65%)	15 (4.10%)
14	13 (3.55%)	12 (3.28%)	18 (4.92%)
15	10 (2.73%)	14 (3.83%)	16 (4.37%)
16	9 (2.46%)	15 (4.10%)	27 (7.38%)
17	8 (2.19%)	16 (4.37%)	18 (4.92%)
18	5 (1.37%)	10 (2.73%)	19 (5.19%)
19	9 (2.46%)	16 (4.37%)	11 (3.01%)
20	5 (1.37%)	13 (3.55%)	12 (3.28%)
21	9 (2.46%)	10 (2.73%)	13 (3.55%)
22	6 (1.64%)	5 (1.37%)	16 (4.37%)
23	7 (1.91%)	7 (1.91%)	9 (2.46%)
24	4 (1.09%)	8 (2.19%)	8 (2.19%)
25	3 (0.82%)	8 (2.19%)	11 (3.01%)
26	2 (0.55%)	9 (2.46%)	9 (2.46%)
27	5 (1.37%)	10 (2.73%)	10 (2.73%)

Table 3: GAD-7 scores frequency.

Score	Oct 2019<br>N (%)	June 2020<br>N (%)	March 2021<br>N (%)
0	19 (5.19%)	6 (1.64%)	7 (1.91%)
1	15 (4.10%)	15 (4.10%)	13 (3.55%)
2	21 (5.74%)	11 (3.01%)	17 (4.64%)
3	21 (5.74%)	10 (2.73%)	15 (4.10%)
4	20 (5.46%)	22 (6.01%)	16 (4.37%)
5	22 (6.01%)	17 (4.64%)	18 (4.92%)
6	12 (3.28%)	13 (3.55%)	15 (4.10%)
7	13 (3.55%)	14 (3.83%)	18 (4.92%)
8	14 (3.83%)	5 (1.37%)	18 (4.92%)
9	35 (9.56%)	9 (2.46%)	15 (4.10%)
10	15 (4.10%)	17 (4.64%)	18 (4.92%)
11	15 (4.10%)	22 (6.01%)	15 (4.10%)
12	33 (9.02%)	27 (7.38%)	12 (3.28%)
13	15 (4.10%)	15 (4.10%)	14 (3.83%)
14	13 (3.55%)	18 (4.92%)	10 (2.73%)
15	10 (2.73%)	16 (4.37%)	11 (3.01%)
16	15 (4.10%)	17 (4.64%)	13 (3.55%)
17	12 (3.28%)	13 (3.55%)	18 (4.92%)
18	17 (4.64%)	10 (2.73%)	25 (6.83%)
19	9 (2.46%)	20 (5.46%)	15 (4.10%)
20	5 (1.37%)	20 (5.46%)	28 (7.65%)
21	15 (4.10%)	49 (13.39%)	35 (9.56%)
